# Integrating Sphere Finger-Photoplethysmography: Preliminary Investigation towards Practical Non-Invasive Measurement of Blood Constituents

**DOI:** 10.1371/journal.pone.0143506

**Published:** 2015-12-04

**Authors:** Takehiro Yamakoshi, Jihyoung Lee, Kenta Matsumura, Yasuhiro Yamakoshi, Peter Rolfe, Daiki Kiyohara, Ken-ichi Yamakoshi

**Affiliations:** 1 Department of Information and Systems Engineering, Faculty of Information Engineering, Fukuoka Institute of Technology, 3-30-1 Wajiro-higashi Higashi-ku, Fukuoka-shi, Fukuoka, 811–0295, Japan; 2 Division of Bioengineering and Bioinformatics, Graduate School of Information Science and Technology, Hokkaido University, Kita 14 Nishi 9, Kita-ku, Sapporo-shi, Hokkaido, 060–0814, Japan; 3 Department of Automatic Measurement and Control, Harbin Institute of Technology, 92 West Dazhi Street, Nan Gang District, Harbin, 150001, China; 4 Graduate School of Natural Science and Technology, Kanazawa University, Kakuma-machi, Kanazawa-shi, Ishikawa, 920–1192, Japan; 5 Showa University School of Medicine, 1-5-8 Hatanodai, Shinagawa-ku, Tokyo, 142–8555, Japan; The University of Tokyo, JAPAN

## Abstract

The aim of this study was to compare conventional photoplethysmography (PPG) in a finger with PPG using an integrating sphere (_IS_PPG) to enhance scattered light collection. Two representative wavelengths were used; 1160 nm, a window through the absorption spectra of water and alcohol, and 1600 nm around where water absorption is high and there is an absorption peak of blood glucose. Simultaneous transmission-type measurements were made with conventional PPG and with _IS_PPG for each wavelength in the tips of index fingers of both hands in a total of 10 healthy young male and female volunteers (21.7 ± 1.6 years old). During a 5 min period in which subjects were in a relaxed state we determined the signal-to-noise ratio, SNR, and the PPG detectability (or sensitivity) by the two techniques. SNR during the test period was significantly higher with _IS_PPG as compared with conventional PPG, especially for the 1600 nm wavelength. PPG signals with 1600 nm could scarcely be detected by conventional PPG, while they could be detected with good sensitively by _IS_PPG. We conclude that under controlled conditions _IS_PPG has better SNR and higher sensitivity than conventional transmission PPG, especially in wavelength regions where water absorption is high but where there is potential for practical measurement of blood constituents including glucose.

## Introduction

Photoplethysmography [1, 2], specifically finger-photoplethysmography, is a useful non-invasive technique for the measurement of a number of important physiological indices that can be derived from the signal produced; the photoplethysmogram (PPG). The primary application of the technique has been the detection of the peripheral blood volume pulse, the so-called alternating-current (AC) component of the PPG, which then allows the calculation of heart rate or, more correctly, pulse rate (e.g., [3–5]). The PPG also contains a so-called direct-current (DC) component, which, at a simplistic level, has been considered to reflect the relatively constant light attenuation by the tissue segment being interrogated. In fact, this DC component has attracted more attention and study, which led to the identification of very low frequency components that are associated with specific physiological processes. As the origin and basis of the AC and DC components of the PPG became better understood the method began to be used for other purposes, in physiological research and clinical applications. These have included: the measurement of blood pressure using the volume-oscillometric principle [6–9]; beat-by-beat blood pressure using the volume-compensation principle [7, 10]; blood pressure sensor from pulse transit time [11]; peripheral arterial stiffness [12]; pulse volume and normalized pulse volume as an indication of peripheral vascular tone [3, 13–15]; skin blood flow [16, 17]; blood oxygen saturation [18, 19]; and levels of blood constituents including blood alcohol and glucose based on near infrared (NIR) spectroscopy [20, 21]. In all of these applications there is a continuing need for improved precision and accuracy (or signal-to-noise ratio) as well as sensitivity (or detectability) in the measurement of the finger-photoplethysmogram (FPPG). This is particularly the case in the attempts to measure blood constituents non-invasively using multiple wavelength photoplethysmography. Research work in this field is still in its infancy in terms of the technology required to achieve the necessary measurement sensitivity, precision and accuracy, and it has been reported that ultrahigh precision measurement of the PPG is essential if valid practical use is to be achieved [20, 22]. There are several factors that influence the sensitivity, precision and accuracy of PPG and they must all be taken into account when attempting to achieve improvements.

The interaction of light with biological tissue is complex and includes the optical processes of multiple scattering, absorption, reflection, transmission and fluorescence [23], and these processes are also influenced by the wavelength of the interrogating optical radiation [24, 25]. Although recent studies demonstrated some benefits from the use of green light (wavelength around 530 nm) PPG in ambulatory settings [4, 26, 27], the red or near-infrared (NIR) wavelengths are often chosen for the PPG light source. The geometrical arrangement of the source and detector is also important and there are two main configurations suitable for FPPG: firstly, transmission-type where the fingertip is placed between the light source and detector and, secondly, reflection-type where the source and detector are placed side-by-side on the tissue surface. Clearly the relative positions of the source and detector are key determinants of the photon paths, which, along with the scattering characteristics of the tissue, set the effective pathlength. The photon paths in relation to discontinuities in the interrogated tissues, such as soft tissue/bone interfaces and both micro-vasculature and large blood vessels, are critical. This is because these photon paths will determine the magnitude of the pulsatile component of the PPG that results from cardiac contraction-related blood volume pulsations that constitute the AC component of the PPG. The pathlength also influences the DC component of the PPG and this is subject to influences by breathing, sympathetic nerve activity and by movement artifact.

When considering the matter of the sensitivity (detectability), specificity, precision and accuracy of photoplethysmographic measurements the fundamental mechanisms of absorption and scattering that pertain with either transmission-type or reflection-backscatter-type require examination. In both cases, the resulting FPPG incorporates only a fraction of the photons undergoing multiple scatter that propagate by transmission or reflection through the tissue segment, as many photons escape capture. The question therefore arises, if it were possible to capture efficiently a larger number of photons emerging from the tissue segment under interrogation would the FPPG signal be enhanced in terms of the sensitivity (detectability) of measurement and, if so, would this also enhance the specificity, precision and accuracy? In order to address this question we therefore propose and establish here a new technique for measuring the FPPG using an integrating sphere to enhance photon collection; integrating sphere finger-photoplethysmography, _IS_FPPG.

The integrating sphere is a very useful optical component for collecting photons that are propagating in multiple directions, for example as they emerge from highly scattering media, which is the case in photoplethysmography. In the conventional use of an integrating sphere photons from the sample under examination would enter the input port of the integrating sphere and would subsequently be uniformly scattered in all directions at the highly reflecting internal surface of the sphere. In an ideal case the photons appearing at the outlet port of the integrating sphere would represent the integral of the multi-directional photon stream emerging from the sample. In such a case the optical gain achieved arises from the collection of almost the whole 2π steradians of available photons from the hemispherical surface of the sample. If we translate this process to the case of a finger being interrogated by incident light then there is a question about the configuration of an integrating sphere that would be practical to use yet still able to achieve highly efficient photon capture.

Our hypothesis is that, compared with conventional photoplethysmographic apparatus, the integrating sphere-type photoplethysmograph, when used for measurements in a finger, would have higher signal-to-noise ratio and detectability than conventional photoplethysmographs. We particularly focus on the transmission-type configuration in this study, by reason, firstly, of its wide utilization in clinical and research situations and, secondly, because it allows a similar alignment of a light source and a photodetector between the two types being compared here. If this hypothesis were to be proven then it could have breakthrough implications in the development of apparatus for non-invasive blood constituent measurement. In this preliminary study, we therefore developed photoplethysmographic apparatus incorporating an integrating sphere to test this hypothesis. We used two particular NIR wavelengths with laser diodes (LD) as light sources: (1) 1160 nm was used as an NIR wavelength situated in a window in the absorption spectra of water and (blood) alcohol; and (2) 1600 nm as an NIR wavelength for which, firstly, there is strong absorption by water which creates considerable challenges for *in vivo* measurement and, secondly, which has potential for blood glucose measurement. This paper describes the fundamental performance of this integrating sphere photoplethysmography system when used for measurements in the index finger and compares the results with those derived from a conventional photoplethysmographic system applied to the contra-lateral index finger.

## Materials and Methods

### System description

**Fig 1:**
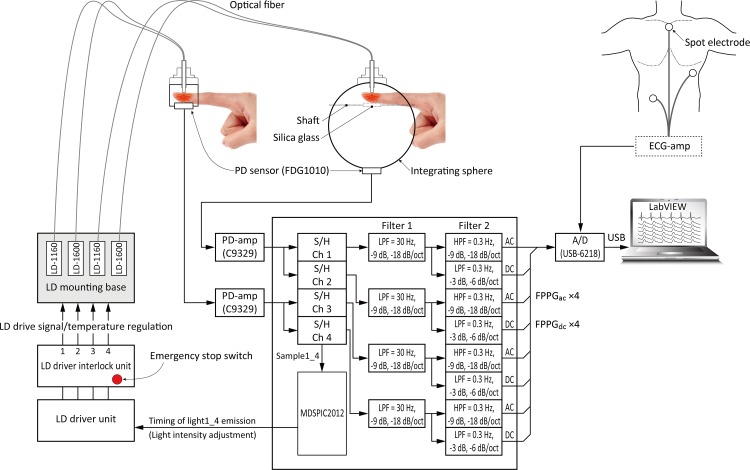
Outline of experimental setup. Experimental setup for the measurement of finger-photoplethysmograms using a conventional transmition-type configuration and an integrating sphere-type with NIR laser light at two wavelengths. (See text for more detailed explanation.) *AC* = alternating-current component, *DC* = direct-current component, *ECG* = electrocardiogram, *FPPG* = finger-photoplethysmogram, *HPF* = high pass filter, *LD* = laser diode, *LPF* = low pass filter, *PD* = photo diode, *S/H* = sample and hold.

**Fig 2:**
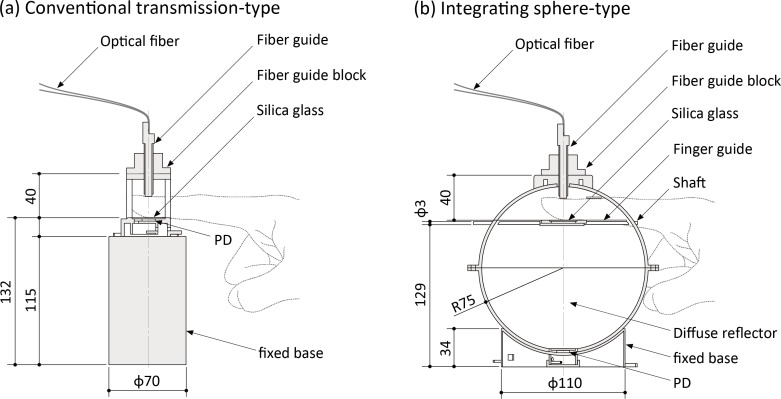
Schematic drawings of the finger jigs and dimensions (mm). (a) conventional transmission-type, and (b) integrating sphere-type. *PD* = photo diode

Fig 1 shows an outline of the complete experimental system for the measurement of FPPGs. The system consists of four main sections: (1) a control section; (2) an LD light source section; (3) a sensing section; and (4) a monitoring and recording section. By applying a time-sharing transmission arrangement and associated synchronous demodulation the system can simultaneously produce four FPPG signals, two for each wavelength, each comprising of a direct-current (DC) component (FPPG_dc_) and an alternating-current (AC) component (FPPG_ac_). FPPGs for each of the two wavelengths are simultaneously obtained from two types of finger jig constructed in our laboratory, one being a conventional transmission-type and the other an integrating sphere-type, as shown in Fig 2. The inner surface of the integrating sphere and all other internal parts of the sphere are coated with white diffuse reflector paint (#83–890, Edmond Optics Japan Inc., Japan), which yields absolute reflectance >92% from 250 nm to 1700 nm.

The LD light source section is comprised of three units: 1) an LD driver unit (TXP5016, Thorlabs Inc., NJ, USA); 2) an LD driver interlock unit; and 3) an LD mounting base unit. The LD light is activated by the light timing signals from the control section. We use two wavelengths, 1160 nm (APLD-1160-S20, Alfa Photonics Inc., Riga, Latvia: Spectral width 10 nm) and 1600 nm (APLD-1600-S10, Alfa Photonics Inc., Riga, Latvia: Spectral width 7 nm) and the light is introduced to each sensing section through a custom-made fiber. The specification of this was: 2 m long, step index fiber, core thickness 105 μm and clad thickness of 125 μm, coated to 0.9 mm diameter. The part number is: FC(8F)/SI105-2m/pigtail coated 0.9 mm, and this was made for us by Keystone International Co., Ltd., Chiba, Japan.

The control section initiates four streams of laser light pulses (two at 1160 nm and two at 1600 nm) by sending a series of four channels of timing signals to the LD driver unit. The light collected from the two photoplethysmograph systems (conventional and integrating sphere-based) is detected by photodiodes (PDs: FDG1010; Thorlabs Inc.; NJ; USA). The 4 channels of analogue PPG signals are separately extracted through the sample-and-hold circuits, which, again, are fed with timing pulses from the control unit. The analogue signals are amplified and separated by filtering using a multiple feedback Butterworth filter, and then the AC and DC components of each PPG are further separated. The DC amplifiers are low-pass with a cut-off frequency of <0.3 Hz (1^st^ order: −3 dB, −6 dB/octave) and the AC amplifiers have passbands of 0.3–30 Hz (3^rd^ order: −9 dB, −18 dB/octave). The analogue signals representing the two channels of FPPG_dc_ and FPPG_ac_, together with the ECG obtained using disposable electrodes connected to a standard bio-amplifier are sampled at a frequency of 1 kHz with a resolution of 16 bits and sent to the analogue-to-digital convertor (USB-6218, National Instruments Japan Corp., Japan), which allows digital filtering to be carried out. We employ two digital filters, both having sharp cut-off characteristics at the selected frequencies using the fast Fourier and inverse fast Fourier transform algorithm. The lower cut-off frequency was set at 0.5 Hz. This was determined on the grounds that the breathing-induced vascular volume frequency components are <0.3 Hz, as mentioned above. The higher cut-off frequency was set at 15 Hz. It is known that the cardiac-related components of the PPG signal have negligible frequency components above 15 Hz [28]. This is to be expected since the AC component of the PPG must have a fundamental frequency equal to heart rate, i.e. around 1.2 Hz in a resting adult, and it is appropriate to include the 2^nd^ and 3^rd^ harmonics at 2.4 and 3.6 Hz to fully represent the AC component. Thus all of the energy related to the signal of interest is found well below 15 Hz. Having decided on the “*Signal*” upper frequency cut-off we then classify as “*Noise*” anything present above 15 Hz. In the present study we arrange for the volunteers to be in a resting state in the darkened test room. This ensures that movement artifacts and interference from natural or artificial light may be regarded as having been essentially eliminated entirely, or at least minimised. Any remaining noise is, therefore, instrument noise from the photodetector, the amplifiers and also possibly from laser switching, which can be transmitted through the power supplies. The digital data are sent to the monitoring and recording section using LabVIEW2009 software (National Instruments Japan Corp., Tokyo, Japan), of course, to confirm the real time waveform during the experiment.

Based on the ‘Japanese Industrial Standards C 6802 (2005)’, the intensity of the pulsed laser light at each wavelength was limited to a maximum of 7 mW (at the exit of fiber-probe).

### Experimental method

#### Ethics statement

The study safeguards and protocols were approved by the ethics commission of the Faculty of Medicine of Kanazawa University (May 18, 2011, No.11), and the study was performed in accordance with the ethical standards laid down in the 1964 Declaration of Helsinki and its later amendments. All subjects agreed to take part in the study voluntarily and signed an informed consent statement.

#### Participants

A total of 10 healthy young male (= 5) and female (= 5) participants, with a mean ± standard deviation age of 21.7 ± 1.6 years, participated in the study. Because no effect size is available from previous studies, the sample size *n* = 10 was determined arbitrarily. Inclusion criteria were having no history of or current cardiovascular disease, and not taking any prescription medication. Participants were requested to refrain from any medication for at least 1 day before the experiment, and from smoking, intense physical activity, eating, alcohol, and taking caffeine-containing substance for least 2 hours before.

#### Procedure and experimental design

The experiment was conducted in a quiet, darkened experimental room having restricted-entry, controlled at a temperature and humidity of approximately 25°C and 50%, respectively. A desk and a comfortable chair for participants to sit on were made available in this room. The measuring sections of the FPPG from the integrating sphere-based system and the conventional system were placed on the right and left side of the participant and kept at heart level. The spot electrodes required for reference lead II electrocardiogram (ECG) measurement were attached to the chest. At the start the participants sat on a chair to relax, and then both index fingers were inserted and fixed into the measuring sections. The stages of the experiment were carried out in the following order while the participants sat quietly: (a) adaptation for 10 min; (b) rest measurement for 5 min. After this there was a 10 min rest, then the same order of experiment was repeated by swapping over the measuring sections to counterbalance any bilateral differences that might be present. The test was performed over a 40 min period and at the same time of day (14:00 p.m.) for each participant.

#### Definition of signal-to-noise ratio and sensitivity

The performance characteristics of the two FPPG systems were compared on the basis of two metrics of signal-to-noise ratio (SNR) and sensitivity (or detectability; the word “sensitivity” is used hereinafter), as defined later. It is known that an idealized PPG contains the DC component and the AC component and both of these, therefore, must be considered in the comparison study. In reality the DC component itself also contains non-DC elements, an important example being the low frequency (approximately 0.3 Hz) signals produced by breathing-induced vascular volume changes. Of course this frequency component depends on breathing and sympathetic nerve frequency and is therefore subject to variation. However, in the present study we only employ the AC components of the two simultaneously recorded FPPGs to compare SNR of the two FPPG systems. To achieve this we must consider and define what we regard as ‘sensitivity’ and as ‘SNR’. Simplistically, sensitivity will indicate the size of the AC components whilst SNR will relate this size to a measure of what we define as ‘noise’. In reality this approach is difficult to implement because PPGs are not absolute quantitative measures, and it is well known that PPGs recorded simultaneously at different anatomical locations will differ in magnitude due to the different absorption and scattering coefficients of the different tissue segments being interrogated as well as differences in the tissue structure and vascularity. Despite this general understanding of the probable lack of anatomical comparability there is actually good evidence to show the similarity of left and right PPGs recorded from the ears, thumbs and toes [1, 29]. Furthermore, it has also been shown that normalizing the AC component against the DC component, as AC/DC, termed the normalized pulse volume [15], does allow comparisons to be made, albeit with caution and specific justifications.

**Fig 3:**
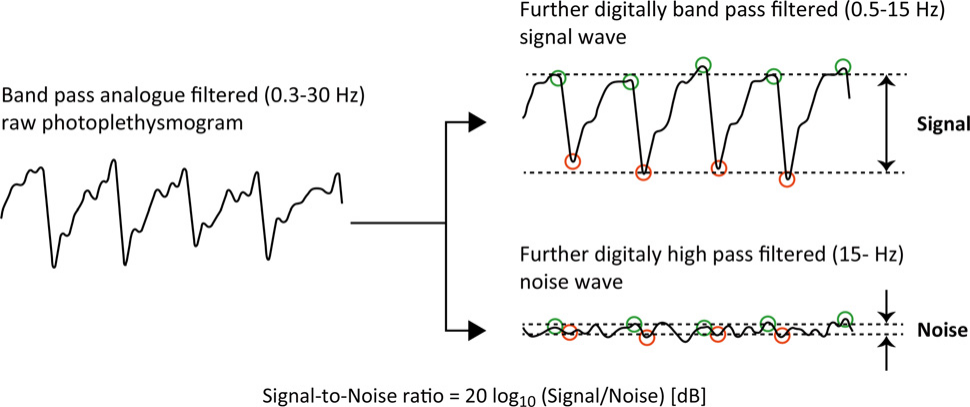
Calculation of the signal-to-noise ratio. This shows how the PPGs are analysed to calculate the signal-to-noise ratios of the two photoplethysmography systems. Note that the original raw analogue filtered signal (0.3−30 Hz) is further digitally filtered (0.5−15 Hz) to produce the ‘*Signal*’ component. Also, the same raw signal is further digitally high pass filtered (15−30 Hz) to produce the ‘*Noise*’ component.

**Fig 4:**
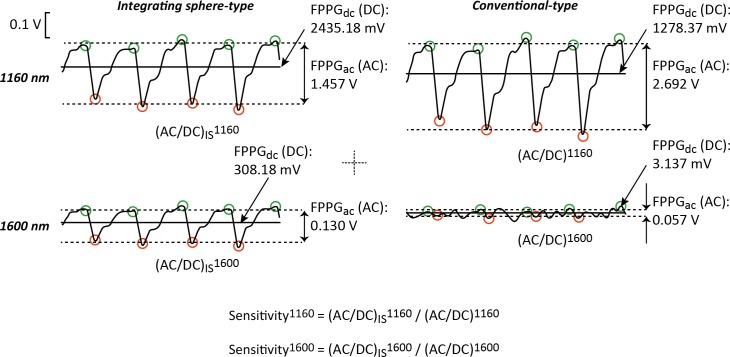
Normalized pulse volume for the each condition. Here the derivation of the normalized pulse volume for the two photoplethysmogram systems for each of the two wavelengths is depicted. *AC* = alternating-current component, *DC* = direct-current component, *FPPG* = finger-photoplethysmogram.

To evaluate the magnitude of the AC components of the PPGs for calculation of the SNRs, beat-by-beat foot and peak points were determined off-line using a Mac computer port of an automated detection program developed by us (see Fig 3). These foot and peak values of the AC components were determined for each of the two interrogating wavelengths using a standard paired local minimum and maximum search algorithm with the parameters of minimum minimum-to-maximum amplitude, maximum minimum-to-maximum interval, and non-detecting time along the time axis. Then the SNR value was calculated from the raw (analogue filtered) PPG waveforms using the following formula:

$$SNR=20\;{\text{log}}_{10}\;\left(\frac{Signal}{Noise}\right)\;\left[\text{dB}\right],$$ (1)

where “*Signal*” is derived as the foot-to-peak amplitude of the 0.5–15 Hz digital band pass filtered raw analogue FPPG signal, and “*Noise*” is derived as the relatively-small irregular amplitude of the 15 Hz digital high pass filtered raw analogue FPPG signal. Even though the foot-to-peak could not be clearly visualised in the high pass FPPG noise component, we nevertheless estimated the noise amplitude by again using the automated detection program mentioned above. The beat-by-beat values of foot-to-peak signal amplitude and noise amplitude were averaged during 300 s of the whole experimental period. In order to determine the sensitivity we used the values of the normalized pulse volume (FPPG_ac_/FPPG_dc_) derived from the raw (analogue filtered) FPPG signals (see Fig 4). We then simply calculated the ratio of the normalized pulse volume values for the conventional transmission-type and the integrating sphere-type (_IS_FPPG), for each wavelength (1160 nm and 1600 nm), using the following formula:

$$Sens^{\lambda }=\frac{A_{IS}^{\lambda }}{A^{\lambda }}\;\left[\;-\;\right],$$ (2)

where A^λ^ and A_IS_^λ^ are derived as the normalized foot-to-peak amplitude (FPPG_ac_/FPPG_dc_) of the raw FPPG and _IS_FPPG signals for each wavelength (1160 nm and 1600 nm), respectively.

The analysis of averaging procedure and period were the same as for the calculation of SNR mentioned above.

#### Data reduction and analyses

SNR values for each wavelength (1160 nm and 1600 nm) and condition (detection technique: integrating sphere-type and conventional transmission-type) were analysed using a series of separate two-way repeated-measures analysis of variance (ANOVA). The Greenhouse-Geisser correction was applied to the degree of freedom where appropriate. For post-hoc comparison, tests for simple main effects were used. These statistical analyses were carried out using IBM SPSS Statistics 18 for Windows (IBM Inc., Tokyo, Japan).

In addition, to consider our definitions of “*Signal*” and “*Noise*” in context, a spectral analysis was carried out on some typical ‘raw analogue FPPG signals’. The power spectra of these FPPGs were calculated using the fast Fourier transform with BIMUTUS II (Kissei Comtec Inc.,Tokyo, Japan).

## Results

### Sample recordings of FPPGs

**Fig 5:**
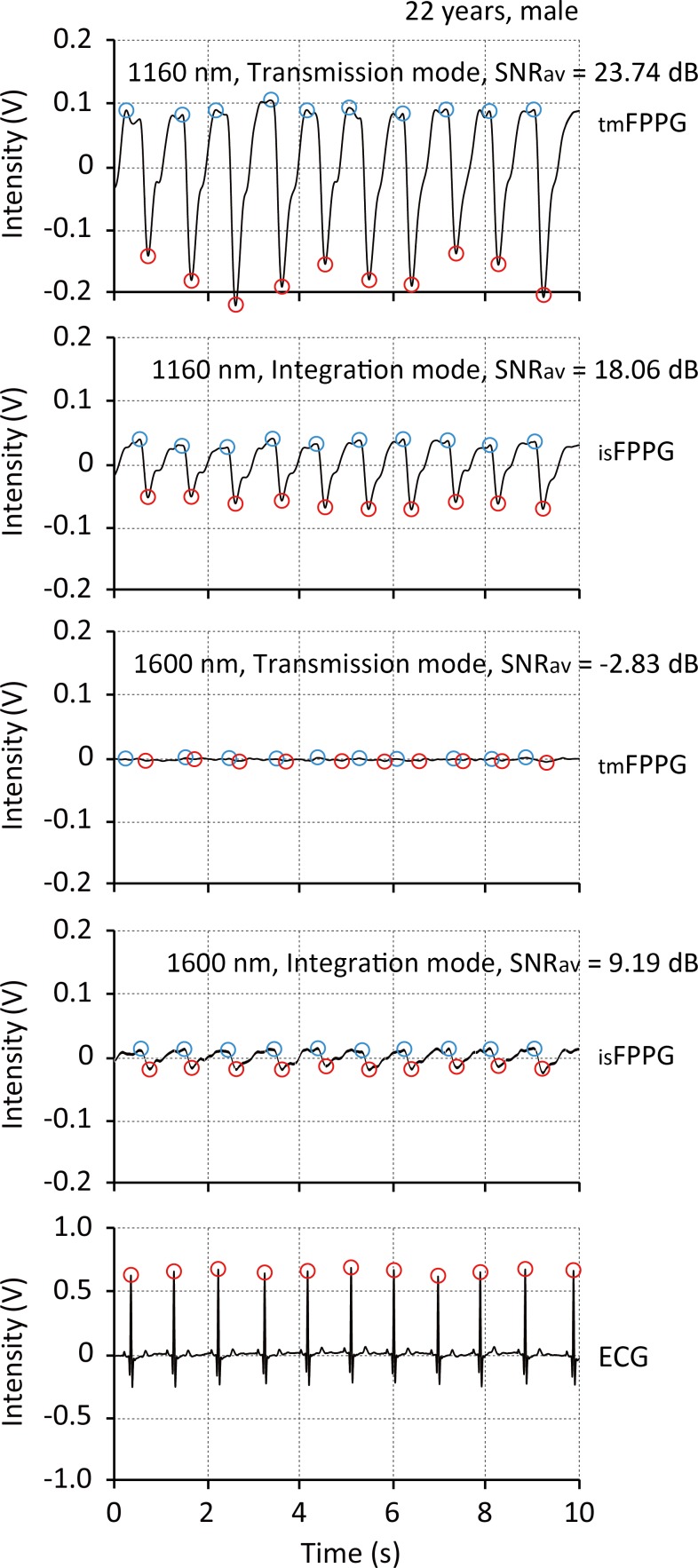
Sample recordings of finger-photoplethysmogram. Typical example recordings of simultaneous measurements by the conventional transmission-type and integrating sphere-type finger-photoplethysmograph with 1160 nm (upper two records) and with 1600 nm (middle two records) light sources, together with the ECG for reference of cardiac beatings (lowest record). *ECG* = electrocardiogram, *SNR*_*av*_ = average value of signal-to-noise ratio during 5 min of cardiac contractions.

Fig 5 shows a typical simultaneous recording of the AC components of the four FPPGs (0.5–15 Hz digital band pass filtered) derived from the conventional transmission-type FPPG and the integrating sphere-type (_IS_FPPG) with 1160 nm (upper two records) and with 1600 nm (middle two records), together with the ECG for reference of cardiac contraction (lowest record). The chart clearly shows the pulsatile FPPG waveforms (AC components) derived from both the conventional transmission-type and integrating sphere-type at 1160 nm, and from the integrating sphere-type at 1600 nm, all well synchronized with the ECG R-peaks. Significantly, with the voltage scales used here the PPG pulsatile waveforms are not visible with the conventional transmission-type at 1600 nm.

### Sample power spectra of FPPG

**Fig 6:**
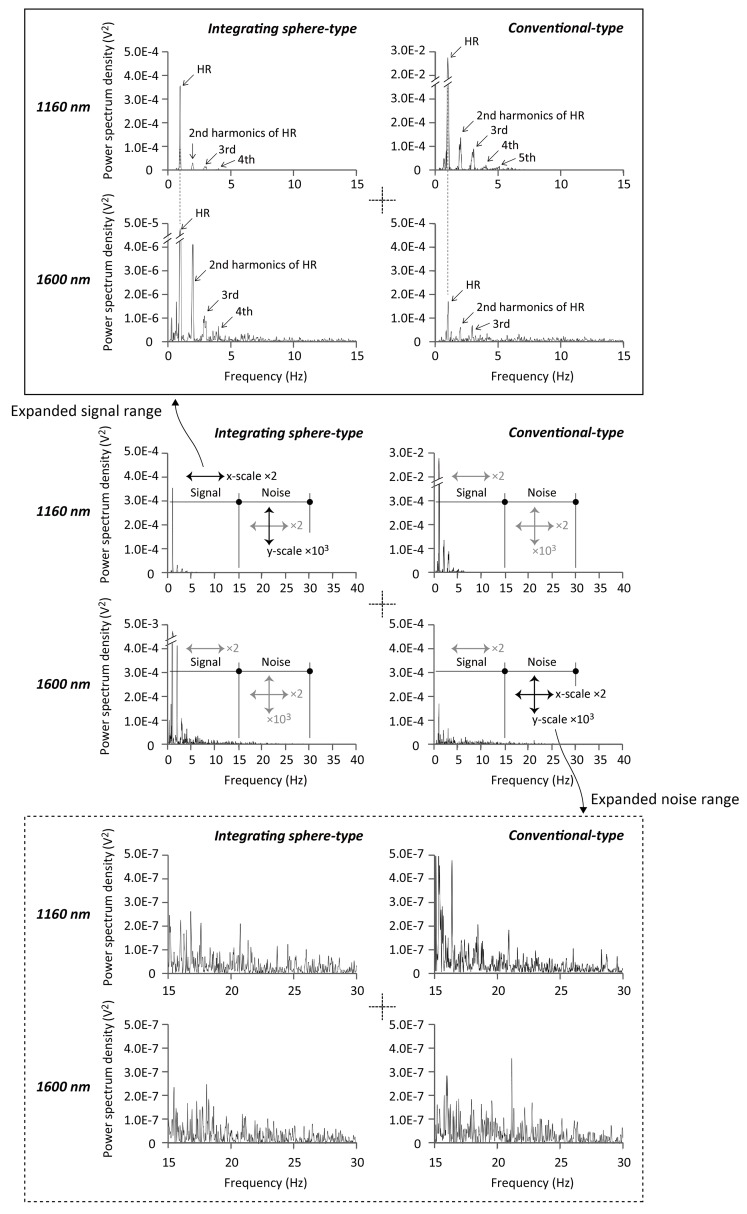
Sample spectra of finger-photoplethysmogram. Typical example of power spectra derived from the photoplethysmograms obtained from the conventional transmission-type instrument (right two spectra) and the integrating sphere-type instrument (left) with wavelengths of 1160 nm (upper) and 1600 nm (lower).

Fig 6 shows a typical example of FPPG power spectra derived from measurements made with the conventional transmission-type instrument (right two spectra) and the integrating sphere-type instrument (left two spectra) with the wavelengths of 1160 nm (upper two spectra) and 1600 nm (lower two spectra). These graphs demonstrate the quantitative power difference between the chosen signal and noise bands for each instrument.

### Signal-to-noise ratio of FPPG

**Fig 7:**
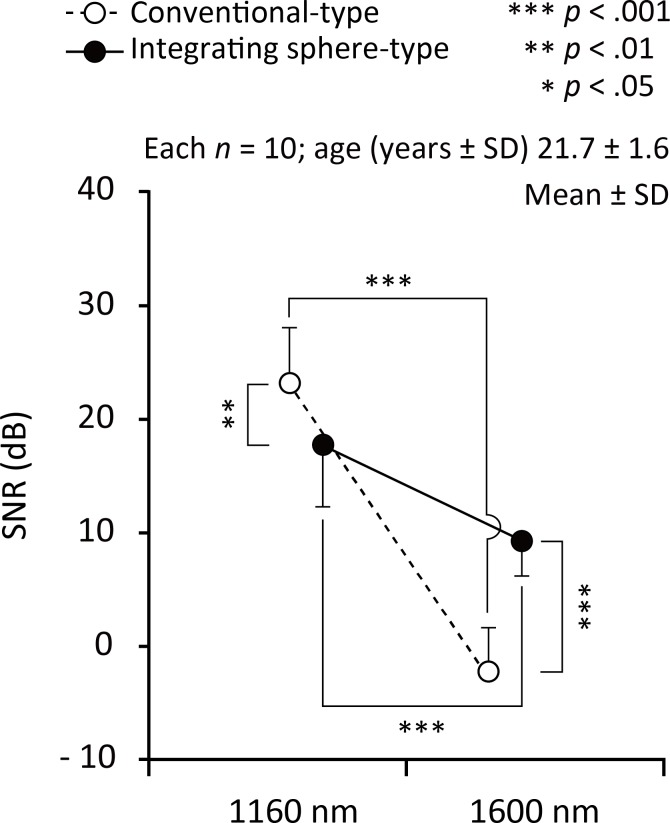
Result of signal-to-ratio of each wavelength. Summarized signal-to-noise ratio (SNR) values under measurement conditions of the conventional transmission- and integrating sphere-type with the wavelengths of 1160 nm (left side) and 1600 nm (right).

Fig 7 shows the summarized results of the signal-to-noise ratio (SNR) values under measurement conditions of the conventional transmission- and integrating sphere-type with the wavelength of 1160 nm and 1600 nm. As for the statistical results of two-way repeated-measure analysis of variance, the interaction between condition (detection technique: conventional transmission- and integrating sphere-type) and wavelength (1160 and 1600 nm) was significant (*F*_1, 36_ = 38.71, *p* < 0.0005, *η*_p_^2^ = 0.518). There was a significant main effect of both condition (*F*_1, 36_ = 4.97, *p* < 0.05, *η*_p_^2^ = 0.121) and wavelength (*F*_1, 36_ = 155.12, *p* < 0.0005, *η*_p_^2^ = 0.812); SNR decreased in conventional transmission-type and 1600 nm. Among the conditions, post hoc tests were significant for conventional transmission-type *vs*. integrating sphere-type for the both 1160 nm (*F*_1, 36_ = 7.97, *p* < 0.01, *η*_p_^2^ = 0.181) and 1600 nm (*F*_1, 36_ = 35.72, *p* < 0.0005, *η*_p_^2^ = 0.498); conventional transmission-type increased SNR significantly more than the integrating sphere-type at 1160 nm, on the other hand, integrating sphere-type increased SNR significantly more than the conventional transmission-type at 1600 nm. Among the wavelengths, post hoc tests were significant for 1160 nm *vs*. 1600 nm for both the conventional transmission-type (*F*_1, 36_ = 174.41, *p* < 0.0005, *η*_p_^2^ = 0.829) and the integrating sphere-type (*F*_1, 36_ = 19.42, *p* < 0.0005, *η*_p_^2^ = 0.350); 1600 nm decreased SNR significantly more than the 1160 nm in the both conditions.

### Sensitivity of FPPG

The sensitivity for the 1160 nm wavelength (*Sens* ^1160^) was 0.43 ± 0.29, and that for 1600 nm (*Sens* ^1600^) was indeterminate because the DC value from the conventional transmission-type system was almost zero, thereby giving an infinite value for AC/DC. This means that PPG signals with 1600 nm could scarcely be detected by the conventional PPG system.

## Discussion

The present study was concerned with the investigation of the use of an integrating sphere to enhance light-collection in finger photoplethysmography. It was hypothesized that instrumentation using the integrating sphere (_IS_FPPG), would be more sensitive and have improved signal-to-noise ratio (SNR) as compared with a conventional transmission-type FPPG instrument. To determine the performance characteristics of the _IS_FPPG we designed an experimental setup to compare the _IS_FPPG with a conventional-type FPPG. We used two infrared diode lasers as essentially quasi-monochromatic radiation sources. One LD had a peak wavelength of 1160 nm, which is where optical absorption by the water component in the tissue is relatively low, i.e. biological window, and where there is an optical absorption band by blood alcohol [20, 22]. The peak wavelength of the second LD was 1600 nm, where very strong optical absorption by the water component dominates [24, 25], but where there is a discernable optical absorption band by blood glucose [21].

The performance metrics used for the comparison were power spectra, SNR and sensitivity. The power spectra provided clear confirmation of the main frequency components of the PPG, which are those dictated by heart rate or, more correctly, pulse rate and its second and third harmonics. It might be argued that, for adults at rest, an adequate bandwidth with which to obtain a faithful record of the PPG AC components could be 0.5–5 Hz. In fact, it has been reported that 0.5–2 Hz could be sufficient [2]. At the same time other authors have reported that, to avoid distortion of the AC component waveform by the differentiating action of filtering, the lower cut-off should be 0.2 Hz [30]. In practical, clinical, applications of photoplethysmography having a lower cut-off frequency at 0.2 Hz would allow breathing to influence the PPG signal and this might be useful if signal processing techniques are then employed to separate cardiac and respiratory components [31]

In this study, we defined 0.5–15 Hz as signal, and 15–30 Hz as noise based on the power spectra as shown in Fig 6.

**Fig 8:**
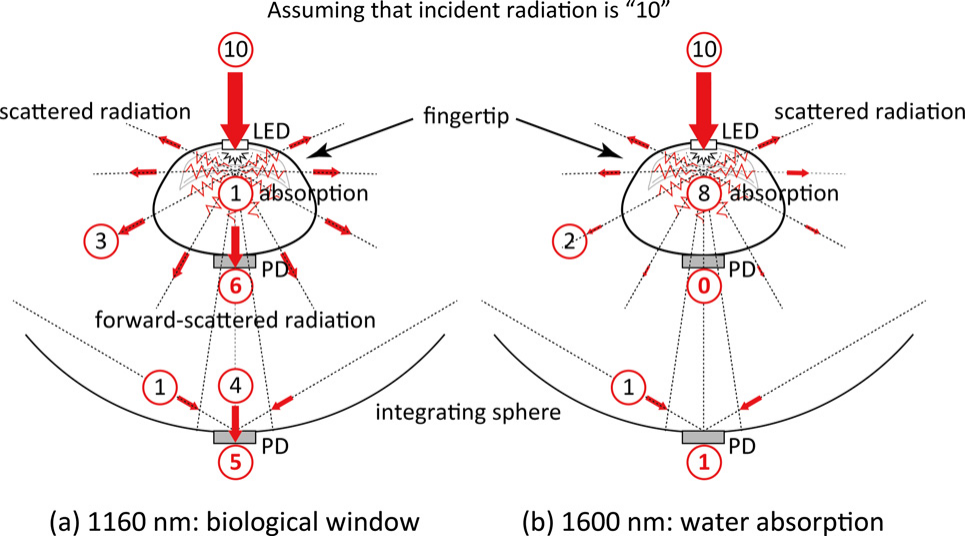
Schematic drawings of possible photon propagation paths in the human finger. (a) 1160 nm, and (b) 1600 nm. (See text for further explanations.) *PD* = photodiode, *LED* = light emitting diode.

The SNR achieved with the 1600 nm wavelength was found to be significantly lower than that for the 1160 nm wavelength and this was the case with both the _IS_FPPG and the conventional FPPG, as indicated in Fig 7. This was also seen in the power spectra in Fig 6. The very strong water absorption at 1600 nm could mean that most of the interrogating incident radiation would be absorbed within the finger tissue, resulting in considerably less photodetection, as schematically illustrated in Fig 8(B). This is also confirmed by the simultaneous recordings shown in Fig 3, where the foot-to-peak points in the FPPG measured by the conventional FPPG were quite unclear due to noise components that were larger than the signal (FPPG), while those by the _IS_FPPG were clearly discriminated with a typical waveform pattern of an FPPG. The SNR values measured by the _IS_FPPG at 1600 nm were, nevertheless, smaller than those at 1160 nm. This definite FPPG acquisition by the _IS_FPPG at 1600 nm is probably mainly due to the integrated photodetection of transmitted and scattered light (see also Fig 8(B)).

The SNR values determined for the conventional FPPG with the 1160 nm wavelength were higher than those for the _IS_FPPG, while those for the _IS_FPPG with 1600 nm were significantly larger than those by the conventional FPPG, as shown in Fig 7. This was also seen in the power spectra (Fig 6). This appears to be an unfavorable result as far as the IS system is concerned. The explanation for this could be related to the fact that at 1160 nm, which is a window in the water absorption spectrum, transmission is higher than at 1600 nm and there is less scatter. This would then simply mean that the light intensity seen by the PD in the conventional FPPG would be greater than that seen at 1600 nm (see Fig 8(A)). As for the integrating sphere system, the light intensity seen by the PD comprises of a direct contribution and a contribution after multiple-scattering. It could be argued that although the magnitude of the direct contribution will have increased as compared with that produced with the 1600 nm wavelength, the integrated total intensity is less that the intensity seen by the PD in the conventional FPPG. This theory would need to be tested by means of optical modelling.

The photodetection sensitivity defined as the ratio of the averaging amplitude of the FPPGs obtained by the integrating sphere and the conventional transmission-type (*Sens* ^λ^ = A_IS_^λ^ / A^λ^) using the 1600 nm wavelength was higher than that using 1160 nm. This could be due to the fact that the waveforms (FPPG) measured by the conventional FPPG with 1600 nm were heavily noise-ridden and their DC and AC values were almost zero in all subjects, indicating that the *Sens* ^1600^ could reach an infinite value.

The first published work on FPPG measurements in the wavelength range having strong optical absorption by the water component in the tissue including 1600 nm was produced by Yamakoshi and Yamakoshi [21]. They reported the development of a very fast spectrophotometer using a halogen lamp as a light source and a liquid nitrogen cooled photodiode-array as a photodetector. This system covered an effective wavelength range from 900 to 1700 nm with a resolution of about 8 nm. This publication proposed “*Pulse Glucometry*” for the non-invasive measurement of blood glucose in the human finger. FPPG measurements in the wavelength range beyond 1350 nm where the strong absorption by the tissue water occurred were clearly demonstrated. These FPPG measurements were made with a bandwidth of 8 nm, and the present measurements by the _IS_FPPG were also successfully carried out using a laser with a full width at half maximum of 7 nm as a light source and a photodiode as a photodetector. As far as we are aware there are no reports concerning such FPPG measurements with a specific wavelength using an integrating sphere photodetection method. As compared with the bulky and costly fast spectrophotometer previously designed, our present method is simpler and more convenient and could serve as a potentially suitable and practically appropriate method for non-invasive optical measurement of blood constituents, including blood glucose, that have distinctive absorption spectra in such a wavelength region.

As for the optical measurement of blood constituents, it should also be noted that other technologies in addition to photoplethysmography have been investigated for more than a decade. These include methods such as Raman spectroscopy [32, 33], surface plasmon resonance spectroscopy [34], polarization [35], photoacoustic spectroscopy [36] and near-infrared diffuse-reflectance spectroscopy [37]. Most of these methods have been under investigation for possible non-invasive measurement of blood constituents but, as yet, there is still no ideal method for practical use.

### Limitations and future work

The trial model of the integrating sphere used in the present study was a one-off, constructed in our laboratories. It is therefore likely that its optical efficiency is less than might be achieved in an ideal case. This could be due to losses at the internal surface, which has less than ideal reflectance. In addition, the positioning of the finger within the sphere is probably less than ideal, which could lead to further losses. To improve the efficiency of the sphere, and to achieve the most suitable arrangement for FPPG measurement, the following issues should be addressed:

1. The most appropriate size of the sphere for the human finger needs to be determined;
2. The positioning of the finger within the sphere for optimum photodetection must be found;
3. Appropriate finger placement within the sphere for stable measurement is needed;
4. Although a transmission-type is widely used method, the integrating sphere FPPG should be compared with a conventional reflectance-type FPPG;
5. The possible influences of skin colour on the performance of the integrating sphere FPPG should be studied.

We are also planning to investigate the development of a multi-wavelength _IS_FPPG system.

## Conclusion

We have described a new optical technique for measuring the finger-photoplethysmogram (FPPG), which we term ‘integrating sphere finger-photoplethysmography’, for non-invasive determination of physiological indices, especially of blood constituents. An experimental laser-based system has been successfully developed for detecting integrating sphere FPPG and conventional FPPG simultaneously with one wavelength in a window of the water absorption spectrum, 1160 nm, and another wavelength where there is strong optical absorption by the water component in the tissue, 1600 nm. We have been able to observe cardiac-related pulsatile changes in optical intensity and we have subsequently been able to detect the FPPG at 1600 nm, a wavelength for characteristic glucose absorption, in 10 healthy adult subjects during 5 min rest period. The results indicate that the technique can detect the FPPG with high sensitivity in the presence of water absorption, and could have potential for practical use in achieving non-invasive optical measurement of blood constituents, as well as other FPPG-based physiological indices mentioned in the Introduction section. Since the present study is a first stage to investigate the new technique, further work is required to determine fully the performance of the integrating sphere finger-photoplethysmograph.
